# A fungicide-responsive kinase as a tool for synthetic cell fate regulation

**DOI:** 10.1093/nar/gkv678

**Published:** 2015-07-02

**Authors:** Kentaro Furukawa, Stefan Hohmann

**Affiliations:** Department of Chemistry and Molecular Biology, University of Gothenburg, Box 462, 40530 Gothenburg, Sweden

## Abstract

Engineered biological systems that precisely execute defined tasks have major potential for medicine and biotechnology. For instance, gene- or cell-based therapies targeting pathogenic cells may replace time- and resource-intensive drug development. Engineering signal transduction systems is a promising, yet presently underexplored approach. Here, we exploit a fungicide-responsive heterologous histidine kinase for pathway engineering and synthetic cell fate regulation in the budding yeast *Saccharomyces cerevisiae*. Rewiring the osmoregulatory Hog1 MAPK signalling system generates yeast cells programmed to execute three different tasks. First, a synthetic negative feedback loop implemented by employing the fungicide-responsive kinase and a fungicide-resistant derivative reshapes the Hog1 activation profile, demonstrating how signalling dynamics can be engineered. Second, combinatorial integration of different genetic parts including the histidine kinases, a pathway activator and chemically regulated promoters enables control of yeast growth and/or gene expression in a two-input Boolean logic manner. Finally, we implemented a genetic ‘suicide attack’ system, in which engineered cells eliminate target cells and themselves in a specific and controllable manner. Taken together, fungicide-responsive kinases can be applied in different constellations to engineer signalling behaviour. Sensitizing engineered cells to existing chemicals may be generally useful for future medical and biotechnological applications.

## INTRODUCTION

All cells respond to external stimuli and execute specific physiological responses through signal transduction systems. Studies in the last two decades have provided detailed knowledge of the molecular mechanisms governing signal transduction systems. Recent advances in synthetic biology have enabled exploiting the signalling components and synthetic genetic circuits for developing novel biological behaviours that could be useful in medicine and biotechnology (1–5). The members of a conserved family of mitogen-activated protein kinases (MAPKs) serve major roles in signal transduction from yeasts to mammals, and mediate responses including altered gene expression, metabolism, secretion, proliferation and apoptosis (6). Hence, customizing MAPK signalling at input and output levels and generating systems that function independently from cell physiology may lead to practical applications such as inhibition of tumour proliferation and induction of apoptosis.

To date, the budding yeast *Saccharomyces cerevisiae* MAPK signalling pathways have been used as a model system to customize their signalling properties with synthetic biological approaches (7). For instance, signalling dynamics of the pheromone-responsive Fus3 and osmoregulatory Hog1 MAPKs can be reshaped by genetic engineering (8–11). These strategies employ synthetic positive or negative regulators artificially converged on MAPK cascades upon their cognate signals (i.e. pheromones or osmotic stress). To reshape MAPK signalling in a more sophisticated way, genetic engineering of upstream sensing systems with orthogonal modules may be a promising approach.

Building artificial biological computations performing Boolean logic gates has recently attracted much attention (12). The logic functions can be used as precise biological switches, so desired combinations of inputs (defined signals) can provide desired outputs (biological behaviours) in a predictable manner. A number of approaches including cell–cell communication with chemical wires (13,14), translational inhibition of transcripts with specific RNA target motifs (15), recombinases that flip the orientation of DNA cassettes (16,17) and designable DNA-binding domains with transcription activator-like repressors (18) have been used to implement logic functions. In addition to tuning signalling dynamics, such efforts may also be useful for regulating desired MAPK outputs at all or none levels.

Common synthetic biological approaches require direct genetic engineering of cells in a certain genetic background, which may require trade-offs in future practical applications. One of the most promising solutions is designing cells able to affect other cells in a specific condition-dependent manner. This kind of approach has previously been demonstrated using engineered *Escherichia coli* cells that sense the microenvironment of a tumour and respond by invading cancerous cells and releasing a cytotoxic agent (19). In another example, transgenic mosquitos (*Aedes aegypti*) were engineered to execute pest control by disseminating a conditional flightless female phenotype among natural populations (20). For the engineering of signal transduction systems, regulated control of a suicide gene that affects signalling in target cells constitutes a promising approach.

Lower eukaryotes use two-component systems as a stimulus sensing module to regulate downstream MAPK signalling cascades. Two-component (also known as phosphorelay) systems do not exist in mammals. The two-component system is composed of a His-Asp phosphorelay from a histidine kinase to a response regulator via a phospho-transmitter. Group III histidine kinases have been reported to be targets of various fungicides such as fludioxonil (Flu) (21). We have recently characterised the group III histidine kinase DhNik1 from the osmotolerant yeast *Debaryomyces hansenii* and obtained dominant, fungicide-resistant mutants (22–24). Although the molecular mechanism of their Flu sensitivity and resistance remains unclear, we realized that the fungicide sensitivity of DhNik1 and its insensitive derivative may serve as synthetic biological tools for engineering fungicide-driven cellular behaviours.

**Figure 1.**
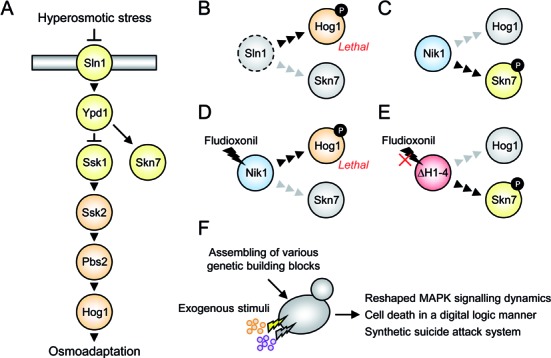
Synthetic cell fate regulation with engineered yeast MAPK signalling. **(A)** Schematic diagram of the yeast Hog1 MAPK pathway consisting of the two-component osmosensor signalling system (Sln1 histidine kinase, Ypd1 phospho-transmitter and Ssk1 response regulator) and the downstream MAPK cascade (Ssk2/Ssk22 MAPKKKs, Pbs2 MAPKK and Hog1 MAPK). The Skn7 response regulator regulates an oxidative stress response via Sln1-Ypd1 and is an alternative and opposite output of the histidine kinase system. Activation of the HOG pathway upon osmotic stress stimulates expression of osmoresponsive genes, while constitutive activation of Hog1 causes lethality. **(B**–**E)** Phosphorylation patterns of Hog1 and Skn7 depend on histidine kinase activity, are affected by Flu treatment in cells expressing DhNik1, but remain unaffected in cells expressing the Flu-resistant DhNik1ΔH1–4 mutant. See the text for more details. Black and grey arrowheads indicate active and silent signalling flows, respectively. **(F)** Genetic engineering of Hog1 MAPK signalling with exogenous stimuli to achieve synthetic cell fate regulation.

In *S. cerevisiae*, the group VI histidine kinase Sln1 regulates the downstream Hog1 MAPK cascade in response to osmotic stress (25,26) (Figure 1A). When the heterologous *DhNIK1* is expressed in *S. cerevisiae*, the DhNik1 protein is able to complement the *sln1* mutation, which otherwise causes lethality because of constitutive activation of Hog1 (27) (Figure 1B, C). Importantly, DhNik1 improperly activates Hog1 upon treatment with Flu even in the presence of Sln1 (Figure 1D), while a DhNik1 mutant (DhNik1ΔH1–4) lacking four of five Histidine kinases, Adenylyl cyclases, Methyl-accepting chemotaxis proteins and Phosphatases (HAMP) domains is not affected by Flu (Figure 1E). Interestingly, DhNik1ΔH1–4 dominantly suppresses the Flu-sensitivity of DhNik1. In this study, we exploited these unique properties of DhNik1 for synthetic cell fate regulation in yeast. Rewiring the Hog1 MAPK signalling achieved three objectives, (i) reshaping the Hog1 MAPK signalling dynamics, (ii) implementing Boolean logic gate like gene expression/cell death and (iii) a synthetic ‘suicide attack’ system (Figure 1F). Our approaches open new avenues for future medical applications such as gene- or cell-based therapies and provide tools for studying MAPK signalling dynamics.

## MATERIALS AND METHODS

### Yeast media and growth conditions

Standard media, synthetic complete (SC; 2% glucose and 0.67% yeast nitrogen base without amino acids supplemented with amino acids to satisfy nutritional requirements) and YPD (1% yeast extract, 2% peptone and 2% glucose) were used for yeast culture and selection of transformants. For growth assays, cells were pregrown overnight on YPD plates, resuspended in water to OD_600_ = 0.1, and 5 μl of a 10-fold dilution series was spotted onto YPD plates with or without chemicals (20 μg/ml Flu, 100 nM Est, 5 μg/ml Dox or a combination of the two). Cell growth was monitored after 24 h culture at 26°C or 37°C.

### Yeast strains and plasmids

Yeast strains, plasmids and primers used in this study are listed in Supplementary Tables S1, S2 and S3, respectively. Details for the construction are described in Supplementary Data.

### Western blot analysis

Yeast cells were grown to mid-log phase in YPD liquid medium. Flu was added to the medium, and 1 ml aliquots were withdrawn at the indicated time points. Cells were resuspended in sodium dodecyl sulphate (SDS) buffer (50 mM Tris-HCl [pH 6.8], 10% Glycerol, 2% SDS, 5 mM NaF, 1 mM Na_3_VO_4_ and 5% β-mercaptoethanol), boiled for 10 min and centrifuged at 13 000 x *g* for 10 min to obtain protein extracts. Protein concentration was measured using the *RC DC* Protein Assay kit (Bio-Rad), and 20 μg of total protein was loaded onto 10% Mini-PROTEAN TGX precast gels (Bio-Rad) and blotted on nitrocellulose membranes (Bio-Rad). Phosphorylated Hog1 was detected using anti-phospho-p38 MAPK antibody (Cell Signaling Technology) with 1:2000 dilution and IRDye 800CW donkey antibody against rabbit IgG (LI-COR Biosciences) with 1:5000 dilution. Total Hog1 protein was detected using anti-Hog1 yC-20 antibody (Santa Cruz Biotechnology, Santa Cruz, CA) with 1:2000 dilution and IRDye 680RD donkey antibody against goat IgG (LI-COR Biosciences) with 1:5000 dilution. Signals were detected using the Odyssey Infrared Imaging System and quantified using the Odyssey 2.1 software (LI-COR Biosciences). All phosphorylated Hog1 values were normalized against the 5-min sample taken from the control strain.

### Reporter assay

**Figure 2.**
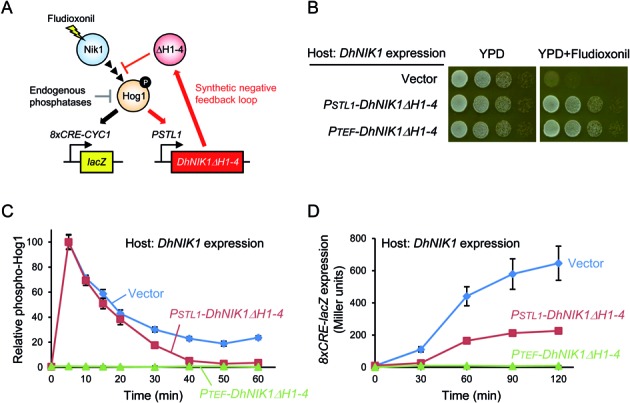
Reshaping HOG signalling dynamics using a synthetic negative feedback loop. **(A)** Heterologous DhNik1 is expressed in yeast cells from its own promoter rendering the HOG pathway sensitive to fludioxonil. A synthetic negative feedback loop was constructed by expressing the fludioxonil-insensitive DhNik1ΔH1–4 from the Hog1-dependent *STL1* promoter. This synthetic feedback downregulates Hog1 in cooperation with the endogenous MAPK phosphatases. The *8xCRE-lacZ* reporter was used to measure output of HOG signalling. **(B)** The synthetic negative feedback as well as constitutive expression of DhNik1ΔH1–4 (*PTEF-DhNIK1ΔH1–4*) confers Flu resistance on yeast cells expressing DhNik1. Cells were spotted on complete medium (YPD) with or without fludioxonil in serial 1:10 dilutions and growth was monitored after 24 h. **(C)** Quantitative Western blotting of relative Hog1 phosphorylation levels. The synthetic negative feedback does not affect the amplitude of Hog1 phosphorylation upon Flu treatment, but more quickly downregulates the phosphorylation level. Western blot images used for quantification are shown in Supplementary Figure S1. **(D)** Reporter gene expression levels as HOG pathway output measure. The synthetic negative feedback mediates lower Hog1-dependent reporter activity than the control. See Materials and Methods for the experimental details.

**Figure 3.**
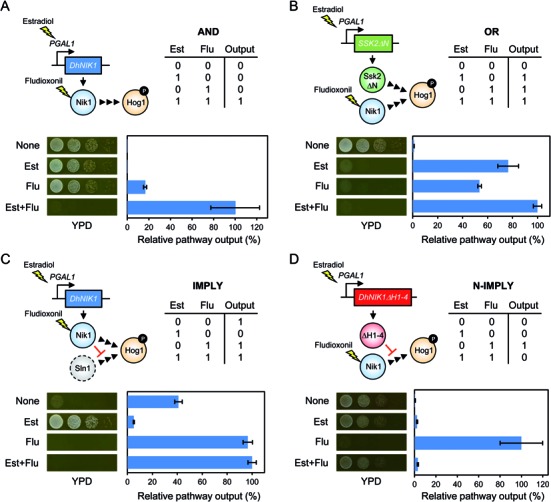
Implementation of simple logic gates employing different means of HOG pathway activation as output. Four yeast strains displaying HOG pathway output as simple Boolean logic functions, **(A)** AND, **(B)** OR, **(C)** IMPLY or **(D)** N-IMPLY gates were constructed by combinatorial integration of genetic parts including *DhNIK1, DhNIK1ΔH1–4* and *SSK2ΔN* under the control of the *DhNIK1* or *GAL1* promoter (with the GEV transcription factor). The truth tables and schematic representations of genes for each logic gate are shown. Pathway output is represented as relative value of the *8xCRE-lacZ* reporter expression (highest reporter activity is set to 100%). Black arrowheads: active signalling flow; yellow lightning: downstream activation effect; red blunted arrows: inhibitory effect. See the text for output details and Materials and Methods for experimental details.

**Figure 4.**
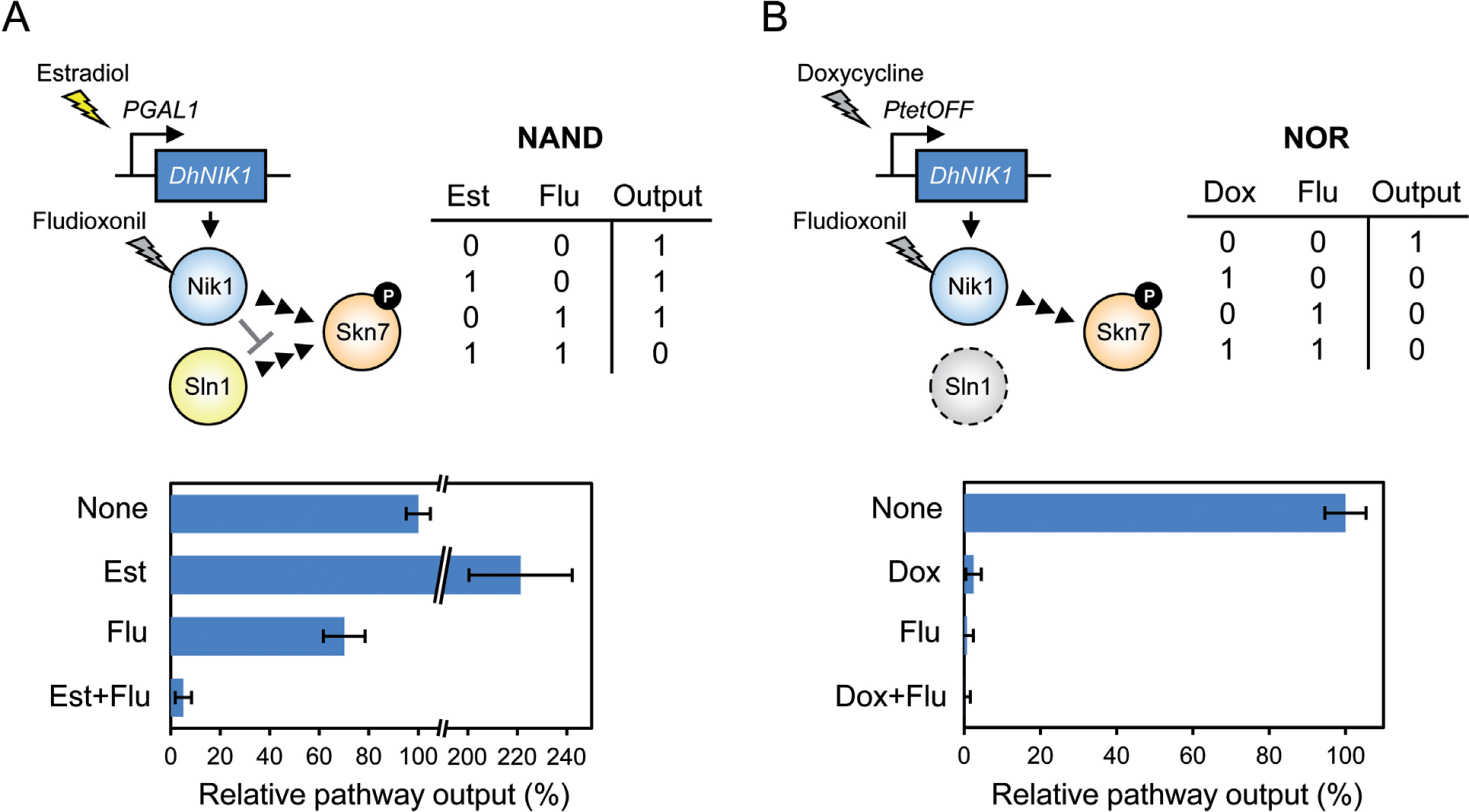
Implementation of NAND and NOR logic gates employing Skn7 as output. Two yeast strains that display Skn7 pathway output as simple Boolean logic functions, **(A)** NAND or **(B)** NOR gate were constructed by control of *DhNIK1* expression under the *GAL1* (with GEV) or *tetO7* promoters, respectively. The truth table and schematic representations of genes for each logic gate are shown. Pathway output is represented as relative value of the *SSRE-lacZ* reporter expression (None is set to 100%). See text for output details and Materials and Methods for the experimental details.

Yeast cells carrying the *8xCRE-lacZ* reporter plasmid were grown to mid-log phase in SC liquid medium lacking histidine, treated with the indicated chemicals for 3 h and then used for reporter assay. Yeast cells carrying the *SSRE-lacZ* or *PFLO11-lacZ* reporter gene were used for reporter assay after grown to OD_660_ = 1.0–1.5 for approximately 14 h in YPD liquid medium with or without chemicals. Specific β-galactosidase activity was measured in cell extracts using the Yeast β-Galactosidase Assay Kit (Pierce) and expressed in Miller units (Figure 2D and Supplementary Figure S4A) or relative values (Figures 3 and 4, Supplementary Figures S2 and S3). Values represent the mean and standard deviation of at least three independent biological replicates.

### Suicide attack assay

Equal amounts (5 μl of OD_600_ = 0.1) of engineered predator and target prey cells were mixed in water and grown on SC plates overnight at 30°C. To determine the fraction of diploid cells formed, the cell mixture was inspected using the Zeiss Axiovert 200 M fluorescence microscope (GFP and mCherry). The cell mixture was resuspended in water to OD_600_ = 0.1, and 5 μl of a 10-fold dilution series were spotted onto YPD plates with or without Flu. Cell growth was monitored after 24 h culture at 30°C or 37°C.

## RESULTS

### Reshaping Hog1 MAPK signalling dynamics with synthetic negative feedback

We examined whether a heterologous histidine kinase could be used to reshape Hog1 signalling dynamics. To this end, we constructed a synthetic negative feedback loop by introducing the gene encoding the Flu-resistant DhNik1ΔH1–4 under the control of the Hog1-dependent *STL1* promoter into yeast cells expressing the Flu-sensitive *DhNIK1* (Figure 2A). In this feedback system, Flu activates Hog1 via inhibition of DhNik1 activity. Hog1 activation results in expression of DhNik1ΔH1–4, which in turn relieves sensor inhibition by Flu. This, then, results in down-regulation of Hog1 activation in cooperation with endogenous MAPK phosphatases. The endogenous expression level of these phosphatases does not seem to be sufficient to down-regulate Hog1 activated by Flu in the engineered signalling system. As expected, the synthetic negative feedback loop conferred Flu resistance on *DhNIK1* expressing cells, as did constitutive expression of *DhNIK1ΔH1–4* (Figure 2B).

To determine the properties of Hog1 signalling dynamics, we monitored Hog1 phosphorylation and *8xCRE-lacZ* activity (28) as fast and slow pathway reporters, respectively. Cells with the synthetic negative feedback initially responded like control cells without feedback. However, after 30 min, the engineered cells showed significant down-regulation of Hog1 phosphorylation (Figure 2C and Supplementary Figure S1). Accordingly, cells with feedback displayed lower *8xCRE-lacZ* reporter activity than the control (Figure 2D). Taken together, these results demonstrate that even in the absence of osmotic stress, Hog1 MAPK signalling dynamics can be reshaped by a synthetic negative feedback with a heterologous system.

### Synthetic control of HOG pathway output

Next we attempted to employ DhNik1 for synthetic control of the HOG pathway output in a Boolean logic gate manner. For this purpose, we used Flu and β-estradiol (Est) as two input stimuli and combined different genetic parts including *DhNIK1, DhNIK1ΔH1–4* and *SSK2ΔN* (a constitutive and hyper-active MAPKKK variant) under control of the *DhNIK1* promoter or the *GAL1* promoter. To be able to activate the *GAL1* promoter with Est, we employed the hybrid transcription factor ‘‘GEV’’ (29), consisting of the Gal4 DNA-binding domain, the human estrogen receptor and the VP16 activation domain. The host strain expresses the temperature-sensitive *sln1-ts4* mutant protein instead of wild-type *SLN1*. This strain shows TRUE behaviour (output is always observed because of constitutive activation of Hog1) at non-permissive temperature and FALSE behaviour (output is always blocked at any condition) in the *hog1Δ* background (Supplementary Figure S2A). We defined the HOG signalling output as both cell death and *8xCRE-lacZ* reporter activation. Moreover, we verified that neither Flu nor Est itself significantly affects the HOG pathway output and cell growth (Supplementary Figure S2B).

We constructed four yeast strains displaying the HOG pathway output as one of four simple Boolean logic functions: AND, OR, IMPLY or N-IMPLY gate (Figure 3). These gates provide different output patterns as illustrated by truth tables. Output of the AND gate cells carrying a *PGAL1-DhNIK1* gene was observed only in the presence of both Est and Flu, where DhNik1 expression is induced by Est and inhibition of DhNik1 activity by Flu causes Hog1 activation (Figure 3A). Output of the OR gate cells carrying *PGAL1-SSK2ΔN* and *PDhNIK1-DhNIK1* genes was observed in the presence of either Est or Flu, where expression of the hyperactive Ssk2ΔN kinase is induced by Est or inhibition of DhNik1 activity by Flu causes Hog1 activation (Figure 3B). Output of the IMPLY gate cells (Sln1 is inactive at non-permissive temperature) carrying the *PGAL1-DhNIK1* gene was not observed in the presence of only Est, where induction of DhNik1 expression by Est inhibits Hog1 activation caused by Sln1 inactivation at restrictive temperature (Figure 3C). In contrast, output was observed under conditions where Hog1 is activated by Sln1 inactivation in the absence of Est or by inhibition of expressed DhNik1 in the presence of both inputs. Finally, output of the N-IMPLY gate cells carrying *PGAL1-DhNIK1ΔH1–4* and *PDhNIK1-DhNIK1* genes was observed in the presence of only Flu, where inhibition of DhNik1 with Flu causes Hog1 activation (Figure 3D). This output was inhibited in the presence of Est because of upregulated expression of the Flu-resistant DhNik1ΔH1–4 variant. Thus, we successfully implemented simple logic gates by rewiring Hog1 MAPK signalling with a few genes and two heterologous inputs.

### Additional Boolean gates employing synthetic control of the Skn7 pathway output

The logic gates presented above were designed such that addition of Flu causes output whenever *DhNIK1* is expressed and *DhNIK1ΔH1–4* is not expressed. This was a limiting factor for the implementation of additional logic gates. To extend the scope of bio-computing, we redesigned DhNik1-based logic gates by employing the Skn7 signalling pathway. The Skn7 transcription factor is an alternative response regulator to Ssk1 in the Sln1-Ypd1-Ssk1/Skn7 signalling module. The Skn7 phosphorylation pattern is opposite to that of Hog1 (Figure 1B–E). To this end, we used Flu and Est or doxycycline (Dox) as input stimuli and expressed *DhNIK1* under the control of the Est-inducible *GAL1* promoter (employing the GEV transcription factor) or the Dox-repressible *tetO7* promoter. Moreover, to focus on the Skn7 signalling output, we used the *SSK1* deletion background to avoid cell death via constitutive Hog1 activation caused by the absence of histidine kinase activity. The *SSRE-lacZ* reporter was used to measure the Skn7 signalling output (23). First, we verified that none of the drugs, Flu, Est or Dox, significantly affected the Skn7 pathway output (Supplementary Figure S3).

We constructed two yeast strains exhibiting Skn7 pathway output as NAND or NOR gate (Figure 4) that could not have been constructed based on Hog1 output. Output of the NAND gate cells carrying the *PGAL1-DhNIK1* gene was not observed in the presence of both Est and Flu, where DhNik1 expression is induced by Est and inhibition of DhNik1 by Flu causes Skn7 inactivation (Figure 4A). Output of the NOR gate cells (in the *sln1Δ* background) carrying the *PtetO7-DhNIK1* gene was not observed in the presence of either Dox or Flu, where DhNik1 expression is repressed by Dox or DhNik1 activity is inhibited by Flu (Figure 4B). Thus, we successfully implemented additional logic gates by rewiring Skn7 signalling with a limited number of genes including the heterologous histidine kinase as well as heterologous inputs.

### Synthetic ‘suicide attack’ system

**Figure 5.**
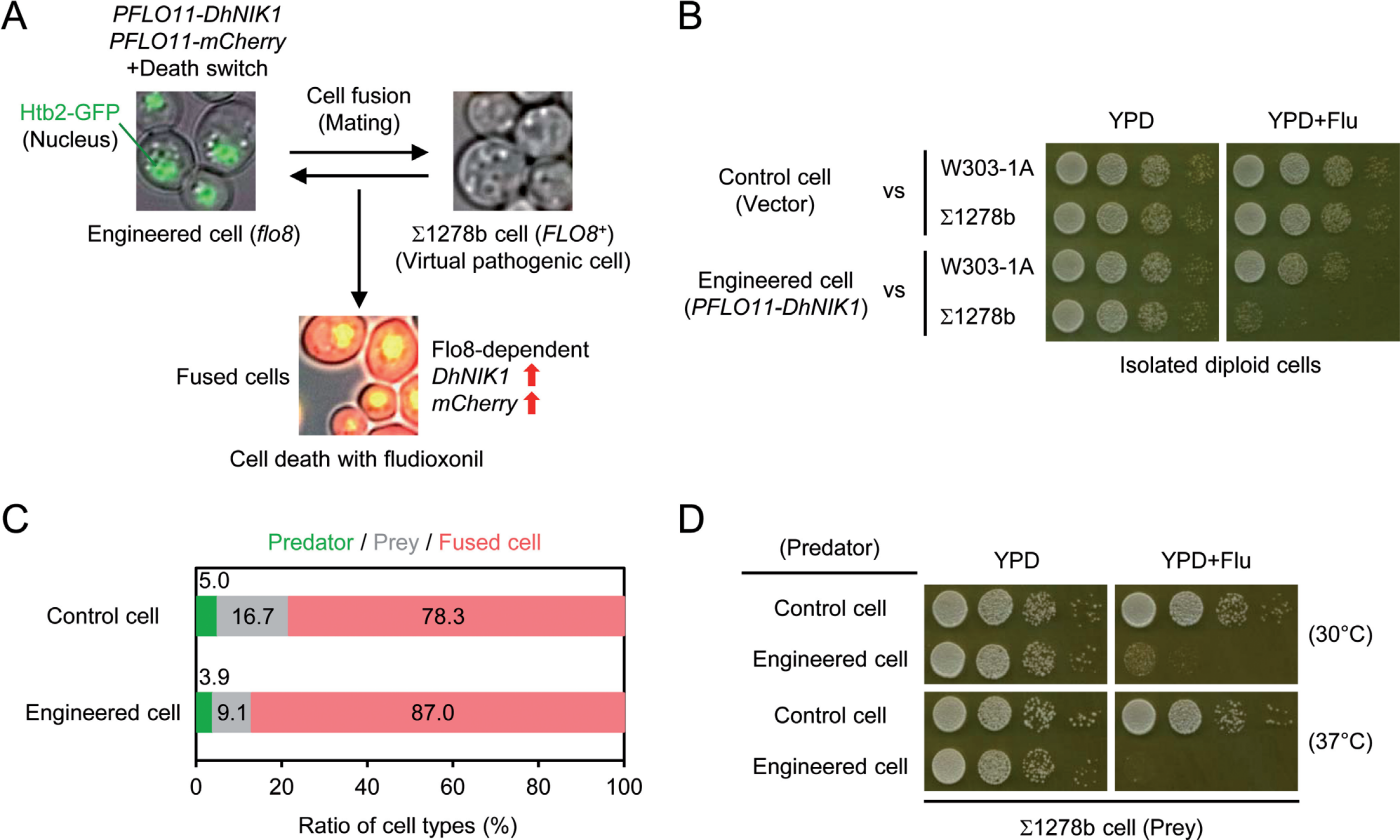
A genetic suicide attack system. **(A)** Concept for the suicide attack system. Engineered *flo8^−^* cells express *DhNIK1* under the control of the Flo8-dependent promoter *PFLO11* and serve as predator cells. When the predator and target Σ1278b *FLO8^+^* prey cells are mixed, they mate/fuse. The resulting diploid cells express DhNik1 and become sensitive to Flu. These three cell types can be distinguished by microscopy (nuclear GFP from engineered cells, no fluorescence from Σ1278b cells and GFP/cytoplasmic mCherry from fused cells). **(B)** Purified diploid cells derived from the engineered predator and Σ1278b prey cells are specifically sensitive to Flu. **(C)** The ratio of predator, prey and diploid cells after cell mixture and incubation for 24 h at 30°C. **(D)** The diploid cells derived from predator and prey cells become sensitive to Flu and growth of excessive predator cells can be controlled by the ‘safety switch’ (temperature sensitive Sln1*-*ts) at 37°C. See Materials and Methods for the experimental details.

We exploited the *DhNIK1* gene for a synthetic predator-prey or suicide attack system, in which engineered cells eliminate themselves taking along target cells in a target cell's genotype- and Flu-dependent manner. The concept is outlined in Figure 5A. In this system the yeast Σ1278b strain serves as a model-target (prey) cell, a virtual pathogen. The Σ1278b background is widely used to study filamentous and invasive growth as well as biofilm formation, phenotypes that are relevant for pathogenicity of yeasts and fungi (30). The engineered predator cells carry the *DhNIK1* and mCherry fluorescent reporter genes under the control of the *FLO11* promoter. Activation of the *FLO11* promoter relies on the Flo8 transcription factor, which is intact in Σ1278b or natural yeast isolates but not in standard laboratory strains (31). Therefore, *DhNIK1* and mCherry are poorly expressed in the engineered laboratory strains, which carry the *flo8^−^* allele. The engineered predator cells can be tracked with a nuclear GFP signal. Predator cells not engaged in trapping can be eliminated by raising the temperature because of the *sln1-ts4* mutation. When the engineered predator cells and target Σ1278b prey cells are mixed, they fuse by mating and form diploid cells, which then contain the genetic information of both predator and prey cells. Hence, those diploid cells are expected to express both DhNik1 and mCherry from the *FLO11* promoter and thereby become sensitive to Flu and distinguishable from the other cell types.

First, we obtained a more tightly regulated derivative of the *FLO11* promoter (2000 bp length) that shows no activity in a *flo8^−^* background and high activity in *FLO8^+^* background (Supplementary Figure S4A). Then, we confirmed that the *PFLO11(2000)-DhNIK1* construct confers Flu sensitivity on Σ1278b haploid and diploid cells, but not on *flo8^−^* cells (Supplementary Figure S4B). We also checked that isolated diploid cells derived from the engineered cells and *FLO8^+^* cells are sensitive to Flu (Figure 5B). Thus, the *PFLO11(2000)-DhNIK1* construct worked properly in a Flo8-dependent manner and the cell survival/lethality pattern can be defined as an AND gate with Flu and Flo8 as two inputs.

We further modified the engineered predator cells by deletion of the *SFL1* gene. Eliminating this transcriptional repressor improves the degree of Flo8-dependency of gene expression. Next, equal amounts of the predator cells and Σ1278b prey cells were mixed and grown overnight for mating on SC plates without any selection. The ratio of the three cell types (non-fused predator cells, non-fused prey cells and fused diploid cells) was determined using fluorescent microscopy (Figure 5C). Around 80% of the cell population was diploid cells. The cell mixture was examined on Flu plates, revealing that the predator cells caused cell growth defects of the total population, in particular at 37°C, where non-mated predator cells do not survive (Figure 5D). These results demonstrate a proof of principle that tightly regulated expression of *DhNIK1* can function as a suicide gene in a specific target cell-dependent manner.

## DISCUSSION

Since development of active chemical compounds to combat diseased or pathogenic cells is a costly and inefficient process, it is desirable to re-use existing, well-characterised drugs or compounds. Recent advances in synthetic biology for engineering signal transduction systems may offer such possibilities. Fungicides are used to avoid infectious diseases by pathogenic fungi. Out of the many known fungicidal targets, the group III histidine kinases have been recognized as targets of the fungicide Flu, and several Flu-resistant histidine kinase mutants have been reported (21). Most work on these kinases has aimed at their identification in various fungi and understanding the molecular mechanism of Flu sensitivity/resistance (23,24). In this study, we demonstrate in three different approaches that the group III histidine kinases and its derivatives are valuable bio-engineering tools for synthetic cell fate regulation.

First, we showed that Hog1 MAPK signalling dynamics following Flu treatment can be reshaped by a synthetic negative feedback loop (Figure 2). Previous work aimed at engineered feedback used the MAPK phosphatase Msg5 or the bacterial effector protein OspF, which directly dephosphorylate MAPKs (9–11). Some of those approaches required genetic knockout of endogenous genes to express synthetic modulators, and the modulators needed to be artificially recruited to the targeted MAPK complex. In contrast, our approach employed controlled expression of an upstream sensing module. In this manner, MAPK signalling dynamics could be reshaped by an orthogonal stimulus rather than by the cognate or endogenous stimuli. Since signal transduction systems frequently have multiple upstream sensing modules, direct tuning of MAPKs may have stronger feedback effects than our approach. However, we anticipate that our feedback system may be useful for artificial control of input levels after a certain time delay, and that engineering upstream and downstream modules together can control MAPK signalling dynamics in an even more sophisticated manner.

Second, we demonstrated Boolean logic gate type cell growth and/or gene expression by combinatorial use of a few genes and chemical inducible/repressible promoters on the basis of the Hog1 MAPK and Skn7 signalling pathways (Figures 3 and 4). Most previous studies on implementation of biological logic gates relied on transcriptional or translational engineering. Our approach is relatively simple because one input (Est or Dox) regulates gene expression and another input (Flu) regulates kinase activity, where the resulting signalling patterns are rewired to the endogenous systems. The digital character of our bio-computing systems was imperfect because the output activity was affected by leaky expression or overexpression of signalling components. Hence, in more advanced systems, levels of gene expression and/or kinase activity should be optimised. Although we could not implement more complex circuits like XOR and XNOR gates, simple gates, which are more suitable for practical applications, were successfully engineered. For instance, the AND gate is potentially useful for artificial control of cell behaviour by expressing various actuators under tight regulation of gene expression with two or more inputs (32–35). Since signalling pathways generally consist of multiple components, multi-layered control of each component may be feasible. In addition, when the DhNik1/Flu system is combined with specific endogenous conditions such as protein and RNA levels (36–38) or when the system is designed as a tightly regulated multiplexer to toggle between the Hog1 and Skn7 signalling pathways, more sophisticated gene circuits can be implemented.

Finally, we presented the genetic-based predator-prey (suicide attack) system, where engineered predator cells carrying the *DhNIK1* gene die in the presence of Flu, taking the Σ1278b prey cells along (Figure 5). Although our engineered yeast cells cannot be used directly for applications, our result demonstrated a proof of principle that tightly regulated expression of *DhNIK1* can function as a suicide gene in a specific target cell-dependent manner. So far, several gene- or cell-based therapies have been conceived for treatment of diseased cells. The general principle of this concept is the use of engineered cells to target specific cells and subsequent killing by well-characterised compounds. For instance, a suicide gene (herpes simplex virus thymidine kinase) and a tumour suppressor gene (p53) can be delivered to cancer cells and used for cancer treatment by eliminating cells with an antiviral drug (ganciclovir) and by suppressing an endogenous p53 mutation, respectively (39). In addition to these straightforward methods, more sophisticated and tightly regulated approaches are required to avoid risks and side-effects, and synthetic biology offers novel methods for gene- or cell-based therapies (40–42). We expect that DhNik1 or other fungicide-responsive systems will be additional useful tools for synthetic biological therapies.

A recent study demonstrated functional transplantations of prokaryotic two-component systems (histidine kinases and chimeric response regulators) into mammalian cells and implementation of two-input logical AND, NOR and OR gene expression (43). To use the approach presented here in practical applications, the sensing module consisting of DhNik1-Ypd1-Ssk1/Skn7 needs to be functionally expressed and integrated into the endogenous signalling systems in host cells. Previous work succeeded in reconstitution of a mammalian MAPK cascade in yeast cells (44). Hence, although direct transplantation of our system into other organisms might be challenging, we may be able to construct and optimize synthetic sensing modules linked with heterologous MAPK cascade in yeast cells and then transplant them into other organisms. Since yeast is an excellent platform for constructing and testing synthetic signalling systems (7), we envision constructing *de novo* signalling systems with DhNik1 in yeast and use those for future applications in medicine and biotechnology.

Controlling the MAPK cascade by a heterologous sensor and a compound and hence independent from the physiological stimulus offers opportunities to study quantitative aspects of the control of MAPK signalling. For instance, the role and regulation of intrinsic feedback mechanisms, the effects of potential artificial feedback loops, control of signalling band width, signalling thresholds and robustness against altering levels or activity of signalling components can now all be studied independent from the physiological stimulus using the tools developed in this work.

## Supplementary Material

SUPPLEMENTARY DATA
