# Supplementary material for: A fungicide-responsive kinase as a tool for synthetic cell fate regulation
Source: Nucleic Acids Res. 2015 Jul 2;43(14):7162–70. doi: 10.1093/nar/gkv678 (PMC4538845; doi:10.1093/nar/gkv678)
Supplement: SUPPLEMENTARY DATA [file supp_43_14_7162__index.html]

A fungicide-responsive kinase as a tool for synthetic cell fate regulation — A fungicide-responsive kinase as a tool for synthetic cell fate regulation — SUPPLEMENTARY DATA 

# A fungicide-responsive kinase as a tool for synthetic cell fate regulation

## SUPPLEMENTARY DATA

- SUPPLEMENTARY DATA
