# Supplementary material for: A fungicide-responsive kinase as a tool for synthetic cell fate regulation
Source: Nucleic Acids Res. 2015 Jul 2;43(14):7162–70. doi: 10.1093/nar/gkv678 (PMC4538845; doi:10.1093/nar/gkv678)

**Supplementary Data**

**A fungicide-responsive kinase as a tool for synthetic cell fate regulation**

**Kentaro Furukawa^1,2,*^ and Stefan Hohmann^1,*^**

^1^Department of Chemistry and Molecular Biology, University of Gothenburg, Box 462, 40530 Gothenburg, Sweden

^2^Present address: Department of Cellular Physiology, Niigata University Graduate School of Medical and Dental Sciences, 1-757 Asahimachi-dori, Chuo-ku, Niigata 951-8510, Japan

^*^To whom correspondence should be addressed.

Tel: +46 31 360 8488; Email: stefan.hohmann@gu.se

Correspondence may also be addressed to Kentaro Furukawa.

Tel: +81 25 227 2158; Fax: +81 25 227 0769; Email: furukawa@med.niigata-u.ac.jp

**SUPPLEMENTARY MATERIALS AND METHODS**

**Construction of plasmids**

Standard DNA manipulation and PCR techniques were performed as described previously (1). Phusion High-Fidelity DNA Polymerase (Thermo Scientific) was used to amplify gene fragments by PCR (primers are shown in Supplementary Table S3). Plasmids used in this study were constructed as follows.

pRS405-DhNIK1: A *DhNIK1* fragment (*Bam*HI-*Xho*I) including its own promoter was excised from pRS423-DhNIK1 (2) and inserted to the same sites of pRS405 (3). The resulting plasmid was linearized by *Bst*EII and integrated into the *leu2* locus.

YIpPSTL1-DhNIK1ΔH1-4: An *STL1* promoter fragment (824 bp with *Kpn*I-*Nco*I) was amplified by PCR using yeast genomic DNA as a template and a *DhNIK1ΔH1-4* fragment (*Nco*I-*Xho*I) was excised from pRS423-DhNIK1ΔH1-4 (2). These fragments were simultaneously inserted into the *Kpn*I-*Xho*I sites of pRS406 (3). The resulting plasmid was linearized by *Stu*I and integrated into the *ura3* locus.

YIpPTEF-DhNIK1ΔH1-4: A constitutive *TEF* promoter fragment (408 bp with *Kpn*I-*Nco*I) was amplified by PCR using pYM-N18 (4) as a template and a *DhNIK1ΔH1-4* fragment (*Nco*I-*Xho*I) was excised from pRS423-DhNIK1ΔH1-4. These fragments were simultaneously inserted into the *Kpn*I-*Xho*I sites of pRS406. The resulting plasmid was linearized by *Stu*I and integrated into the *ura3* locus.

pIH-ADGEV: An *ADH1promoter-GAL4-hER-VP16* fragment (*EcoR*I-*Not*I) was excised from pIT-ADGEV (5) and inserted into the same sites of pRS403 (3). The resulting plasmid was linearized by *Nde*I and integrated into the *his3* locus.

YIpPGAL1-SSK2ΔN: A *GAL1promoter-SSK2ΔN* fragment (*Sal*I-*Sac*I) was excised from pVR91 (laboratory collection) and inserted into the same sites of pRS406. The resulting plasmid was linearized by *Eco*RV and integrated into the *ura3* locus.

YIpPtetOFF7-DhNIK1: A *tetOFF7* cassette (*PCMV-tTA*/*PtetO7* with *Kpn*I-*Nco*I) was amplified by PCR using pCM190 (6) as a template and inserted into the same sites of YIpPGAL1-DhNIK1(T) (7). The resulting plasmid was linearized by *Eco*RV and integrated into the *trp1* locus.

YIpPFLO11(2000/1200)-lacZ: A *FLO11* promoter fragment (2000/1200 bp with *Hin*dIII-*Bgl*II) was amplified by PCR using yeast genomic DNA as a template and inserted into the *Hin*dIII-*Bam*HI sites of YIpMELβ2 (8). The resulting plasmid was linearized by *Nco*I and integrated into the *ura3* locus.

YIpPFLO11(2000)-DhNIK1: A *FLO11(2000)* promoter fragment (*Kpn*I-*Nco*I) was amplified by PCR using yeast genomic DNA as a template and a *DhNIK1* fragment (*Nco*I-*Sac*I) was excised from YIpPGAL1-DhNIK1 (7). These fragments were simultaneously inserted into the *Kpn*I-*Sac*I sites of pRS406. The resulting plasmid was linearized by *Eco*RV and integrated into the *ura3* locus.

YIpPFLO11(2000)-mCherry: First, a *mCherry* fragment (*Hin*dIII-*Bam*HI-*mCherry*-*Bgl*II) was excised from pBS35 (9) and inserted into the *Hin*dIII-*Bam*HI sites of YIplac128 (10), resulting in YIplac128-mCherry (*Bam*HI site in *mCherry* became available). Next, a *FLO11(2000)* promoter fragment (*Hin*dIII-*Bgl*II) was amplified by PCR using yeast genomic DNA as a template and inserted into the *Hin*dIII-*Bam*HI sites of YIplac128-mCherry. The resulting plasmid was linearized by *Eco*RV and integrated into the *leu2* locus.

**Construction of yeast strains**

Yeast transformation was performed as described previously (11). For *HTB2-GFP* expression, an *HTB2-GFP::HIS3* fragment was amplified by PCR using genomic DNA of the BY4741 *HTB2-GFP* strain (EUROSCARF) as a template and integrated into the *HTB2* locus. To activate the *GAL1* promoter with Est, pIT-ADGEV was linearized by *Sna*BI and integrated into the *trp1* locus.

**SUPPLEMENTARY REFERENCES**

1. Sambrook, J. and Russell, D.W. (2001) *Molecular Cloning: A Laboratory Manual* (Cold Spring Harbor Laboratory Press, Plainview, NY), 3rd Ed.
2. Meena, N., Kaur, H. and Mondal, A.K. (2010) Interactions among HAMP domain repeats act as an osmosensing molecular switch in group III hybrid histidine kinases from fungi. *J. Biol. Chem.*, **285,** 12121–12132.
3. Sikorski, R.S. and Hieter, P. (1989) A system of shuttle vectors and yeast host strains designed for efficient manipulation of DNA in *Saccharomyces cerevisiae*. *Genetics*, **122,** 19–27.
4. Janke, C., Magiera, M.M., Rathfelder, N., Taxis, C., Reber, S., Maekawa, H., Moreno-Borchart, A., Doenges, G., Schwob, E., Schiebel, E. and Knop, M. (2004) A versatile toolbox for PCR-based tagging of yeast genes: new fluorescent proteins, more markers and promoter substitution cassettes. *Yeast*, **21,** 947–962.
5. Takahashi, S. and Pryciak, P.M. (2008) Membrane localization of scaffold proteins promotes graded signaling in the yeast MAP kinase cascade. *Curr. Biol.*, **18,** 1184–1191.
6. Garí, E., Piedrafita, L., Aldea, M. and Herrero, E. (1997) A set of vectors with a tetracycline-regulatable promoter system for modulated gene expression in *Saccharomyces cerevisiae*. *Yeast*, **13,** 837–848.
7. Furukawa, K., Randhawa, A., Kaur, H., Mondal, A.K. and Hohmann, S. (2012) Fungal fludioxonil sensitivity is diminished by a constitutively active form of the group III histidine kinase. *FEBS Lett.*, **586,** 2417–2422.
8. Melcher, K., Sharma, B., Ding, W.V. and Nolden, M. (2000) Zero background yeast reporter plasmids. *Gene*, **247,** 53–61.
9. Shaner, N.C., Campbell, R.E., Steinbach, P.A., Giepmans, B.N., Palmer, A.E. and Tsien, R.Y. (2004) Improved monomeric red, orange and yellow fluorescent proteins derived from *Discosoma* sp. red fluorescent protein. *Nat. Biotechnol.*, **22,** 1567–1572.
10. Gietz, R.D. and Sugino, A. (1988) New yeast-Escherichia coli shuttle vectors constructed with *in vitro* mutagenized yeast genes lacking six-base pair restriction sites. *Gene*, **74,** 527–534.
11. Amberg, D.C., Burke, D.J. and Strathern, J.N. (2005) *Methods in Yeast Genetics: A Cold Spring Harbor Laboratory Course Manual* (Cold Spring Harbor Lab Press, Cold Spring Harbor, New York).
12. Tatebayashi, K., Yamamoto, K., Tanaka, K., Tomida, T., Maruoka, T., Kasukawa, E. and Saito, H. (2006) Adaptor functions of Cdc42, Ste50, and Sho1 in the yeast osmoregulatory HOG MAPK pathway. *EMBO J.*, **25,** 3033–3044.
13. Furukawa, K., Sidoux-Walter, F. and Hohmann, S. (2009) Expression of the yeast aquaporin Aqy2 affects cell surface properties under the control of osmoregulatory and morphogenic signalling pathways. *Mol. Microbiol.*, **74,** 1272–1286.

**SUPPLEMENTARY FIGURE LEGENDS**

**Figure S1.** Western blot images used for quantification of Hog1 phosphorylation in Figure 2C. Asterisk bands (*) were used as a positive control in the other membranes. See MATERIALS AND METHODS for experimental details.

**Figure S2.** HOG signalling-specific reporter assay. **(A)** Sln1 inactivation (*sln1-ts4* mutation at 37°C) leads to expression of the *8xCRE-lacZ* reporter, which is completely suppressed by *HOG1* deletion. **(B)** Adding Est, Flu, or both, neither affects yeast cell growth nor *8xCRE-lacZ* reporter expression. Pathway output represents the relative value of *8xCRE-lacZ* reporter expression. See MATERIALS AND METHODS for the experimental details.

**Figure S3.** Skn7 signalling-specific reporter assay. *SSRE-lacZ* reporter expression depends on upstream histidine kinase activity (*SLN1^+^* or *sln1Δ*), but is not affected by Est, Dox, or Flu. Relative pathway output indicates relative value of the *SSRE-lacZ* reporter expression. See MATERIALS AND METHODS for the experimental details.

**Figure S4.** Flo8-dependent gene expression under the control of *FLO11* promoter. **(A)** Deletion analysis of the *FLO11* promoter using *FLO11-lacZ* constructs shows that the 2000 bp promoter shows no activity in *flo8^-^* mutant and high activity in both Σ1278b haploid and diploid strains. **(B)** The *PFLO11(2000)-DhNIK1* construct confers fludioxonil sensitivity on both Σ1278b haploid and diploid strains, but not on *flo8^-^* mutant. See MATERIALS AND METHODS for the experimental details.

**Table S1.** Yeast strains used in this study

Strain Genotype Source

For synthetic negative feedback experiments

YSH1440 *MAT***a** *leu2-Δ1 his3-Δ200 trp1-Δ63 ura3-52 sln1-ts4* Lab collection

YSH2864* YSH1440 *leu2-Δ1::pRS405-DhNIK1 ura3-52::pRS406* This study

YSH2865* YSH1440 *leu2-Δ1::pRS405-DhNIK1 ura3-52::YIpPSTL1-DhNIK1ΔH1-4* This study

YSH2866* YSH1440 *leu2-Δ1::pRS405-DhNIK1 ura3-52::YIpPTEF-DhNIK1ΔH1-4* This study

For logic gate experiments

TRUE* Same as YSH1440 Lab collection

FALSE* YSH1440 *hog1::kanMX* This study

AND* YSH1440 *ura3-52::YIpPGAL1-DhNIK1 trp1-Δ63::pIT-ADGEV* This study

OR* YSH1440 *ura3-52::YIpPGAL1-SSK2ΔN leu2-Δ1::pRS405-DhNIK1* This study

*trp1-Δ63::pIT-ADGEV*

IMPLY* YSH1440 *ura3-52::YIpPGAL1-DhNIK1 trp1-Δ63::pIT-ADGEV (at 37°C)* This study

N-IMPLY* YSH1440 *ura3-52::YIpPGAL1-DhNIK1ΔH1-4 leu2-Δ1::pRS405-DhNIK1* This study

*trp1-Δ63::pIT-ADGEV*

YSH2470 *MAT***a** *leu2-3,112 trp1-1 his3-11,15 ade2-1 can1-100* (7)

*ssk1::LEU2 ura3-1::YIpSSRE-lacZ*

YSH2472 *MAT***a** *leu2-3,112 trp1-1 his3-11,15 ade2-1 can1-100* (7)

*sln1::LEU2 ssk1::kanMX ura3-1::YIpSSRE-lacZ*

NAND YSH2470 *trp1-1::YIpPGAL1-DhNIK1(T) his3-11,15::pIH-ADGEV* This study

NOR YSH2472 *trp1-1::YIpPtetOFF7-DhNIK1* This study

For synthetic suicide attack experiments

W303 ura3-1 *MAT***α** *ura3-1 can1-100 flo8^-^* Lab collection

Σ ura3-52 *MAT***α** *ura3-52 FLO8^+^* Lab collection

CMY77 *MAT***a***/MAT***α** *ura3-52/ura3-52 FLO8^+^/FLO8^+^* Lab collection

YSH2603 YSH1440 *ura3-52::YIpPFLO11(3000)-lacZ* This study

YSH2631 YSH1440 *ura3-52::YIpPFLO11(2000)-lacZ* This study

YSH2604 YSH1440 *ura3-52::YIpPFLO11(1200)-lacZ* This study

YSH2615 Σ ura3-52 *ura3-52::YIpPFLO11(3000)-lacZ* This study

YSH2635 Σ ura3-52 *ura3-52::YIpPFLO11(2000)-lacZ* This study

YSH2617 Σ ura3-52 *ura3-52::YIpPFLO11(1200)-lacZ* This study

YSH2609 CMY77 *ura3-52/ura3-52::YIpPFLO11(3000)-lacZ* This study

YSH2639 CMY77 *ura3-52/ura3-52::YIpPFLO11(2000)-lacZ* This study

YSH2611 CMY77 *ura3-52/ura3-52::YIpPFLO11(1200)-lacZ* This study

YSH2545 YSH1440 *ura3-52::pRS406* This study

YSH2643 YSH1440 *ura3-52::YIpPFLO11(2000)-DhNIK1* This study

YSH2599 Σ ura3-52 *ura3-52::pRS406* This study

YSH2645 Σ ura3-52 *ura3-52::YIpPFLO11(2000)-DhNIK1* This study

YSH2557 CMY77 *ura3-52/ura3-52::pRS406* This study

YSH2647 CMY77 *ura3-52/ura3-52::YIpPFLO11(2000)-DhNIK1* This study

YSH2709 Cross of YSH2545 and W303 ura3-1 This study

YSH2705 Cross of YSH2545 and Σ ura3-52 This study

YSH2706 Cross of YSH2643 and W303 ura3-1 This study

YSH2665 Cross of YSH2643 and Σ ura3-52 This study

YSH2701 YSH1440 *HTB2-GFP::HIS3 leu2-Δ1::YIpPFLO11(2000)-mCherry* This study

*ura3-52::pRS406 sfl1::kanMX*

YSH2700 YSH1440 *HTB2-GFP::HIS3 leu2-Δ1::YIpPFLO11(2000)-mCherry* This study

*ura3-52::YIpPFLO11(2000)-DhNIK1sfl1::kanMX*

* These strains carrying pRS413-8xCRE-lacZ were used for the *8xCRE-lacZ* reporter assay.

**Table S2.** Plasmids used in this study

Plasmid Description Source

pRS403 *HIS3* (3)

pRS405 *LEU2* (3)

pRS406 *URA3* (3)

pYM-N18 *kanMX PTEF* (4)

pRS405-DhNIK1 *LEU2 PDhNIK1-DhNIK1* This study

YIpPSTL1-DhNIK1ΔH1-4 *URA3 PSTL1-DhNIK1ΔH1-4* This study

YIpPTEF-DhNIK1ΔH1-4 *URA3 PTEF-DhNIK1ΔH1-4* This study

pRS413-8xCRE-lacZ *CEN/ARS HIS3 P8xCRE-CYC1-lacZ* (12)

pIT-ADGEV *TRP1 PADH1-GAL4DBD-hER-VP16* (5)

pIH-ADGEV *HIS3 PADH1-GAL4DBD-hER-VP16* This study

YIpPGAL1-DhNIK1 *URA3 PGAL1-DhNIK1* (7)

YIpPGAL1-DhNIK1ΔH1-4 *URA3 PGAL1-DhNIK1ΔH1-4* (7)

YIpPGAL1-SSK2ΔN *URA3 PGAL1-SSK2ΔN* This study

YIpPGAL1-DhNIK1(T) *TRP1 PGAL1-DhNIK1* (7)

pCM190 *2µ URA3 PCMV-tTA PtetO7* (6)

YIpPtetOFF7-DhNIK1 *TRP1 PCMV-tTA PtetO7-DhNIK1* This study

YIpPFLO11(3000)-lacZ *URA3 PFLO11(3000)-lacZ* (13)

YIpPFLO11(2000)-lacZ *URA3 PFLO11(2000)-lacZ* This study

YIpPFLO11(1200)-lacZ *URA3 PFLO11(1200)-lacZ* This study

YIpPFLO11(2000)-DhNIK1 *URA3 PFLO11(2000)-DhNIK1* This study

YIplac128 *LEU2* (10)

pBS35 *hph mCherry* (9)

YIpPFLO11(2000)-mCherry *LEU2 PFLO11(2000)-mCherry*  This study

**Table S3.** Primers used in this study

Primer Sequence (5’ to 3’)

For construction of *PSTL1-DhNIK1ΔH1-4*

P-PSTL1(KpnI)F GGATATGTCTGGTACCCGGCCAAGATAGAATTAAAG

P-PSTL1(NcoI)R ATTTTAAATCCCATGGGGTCTAAAACTTTCTATGTTC

For construction of *PTEF-DhNIK1ΔH1-4*

P-PTEF(KpnI)F ATCCAGTGTCGGTACCGAGCTCATAGCTTC

P-PTEF(NcoI)R GATCCACTAGCCATGGAAAACTTAGATTAGATTGC

For construction of *PtetOFF7-DhNIK1*

P-TetOFF7(KpnI)F TATAGGGCGAATTGGGTACCGAATTCTTATTACGATCCTCG
P-TetOFF7(NcoI)R TCGGGTGTACCCATGGGGATCCCCCGAATTGATC

For construction of *PFLO11(2000/1200)-lacZ/mCherry*

P-PFLO11(-2000+HindIII)F TGCGGAATACAAGCTTATTCTCATCGAGAGCCGAG

P-PFLO11(-1200+HindIII)F GTGCCTGGAAAAGCTTAATTAAGGTTTTTTTCTTC

P-PFLO11(BglII)R CGAGTAGAAAAGATCTTTGCATAGTGTGCGTATATG

For construction of *PFLO11(2000)-DhNIK1*

P-PFLO11(KpnI)F GTGGGTGCTTGGTACCAGTCTTCGTTTCCTATC

P-PFLO11(NcoI)R AATGGTCTTTCCATGGTGTGCGTATATGGATTTTTG

For construction of *HTB2-GFP*

P-HTB2(internal)F CTGAAGCTTCTAAATTGGCCGC

P-HTB2(3-flanking)R CTCCCTTCGGAGGCTGATAG

**Figure S1**


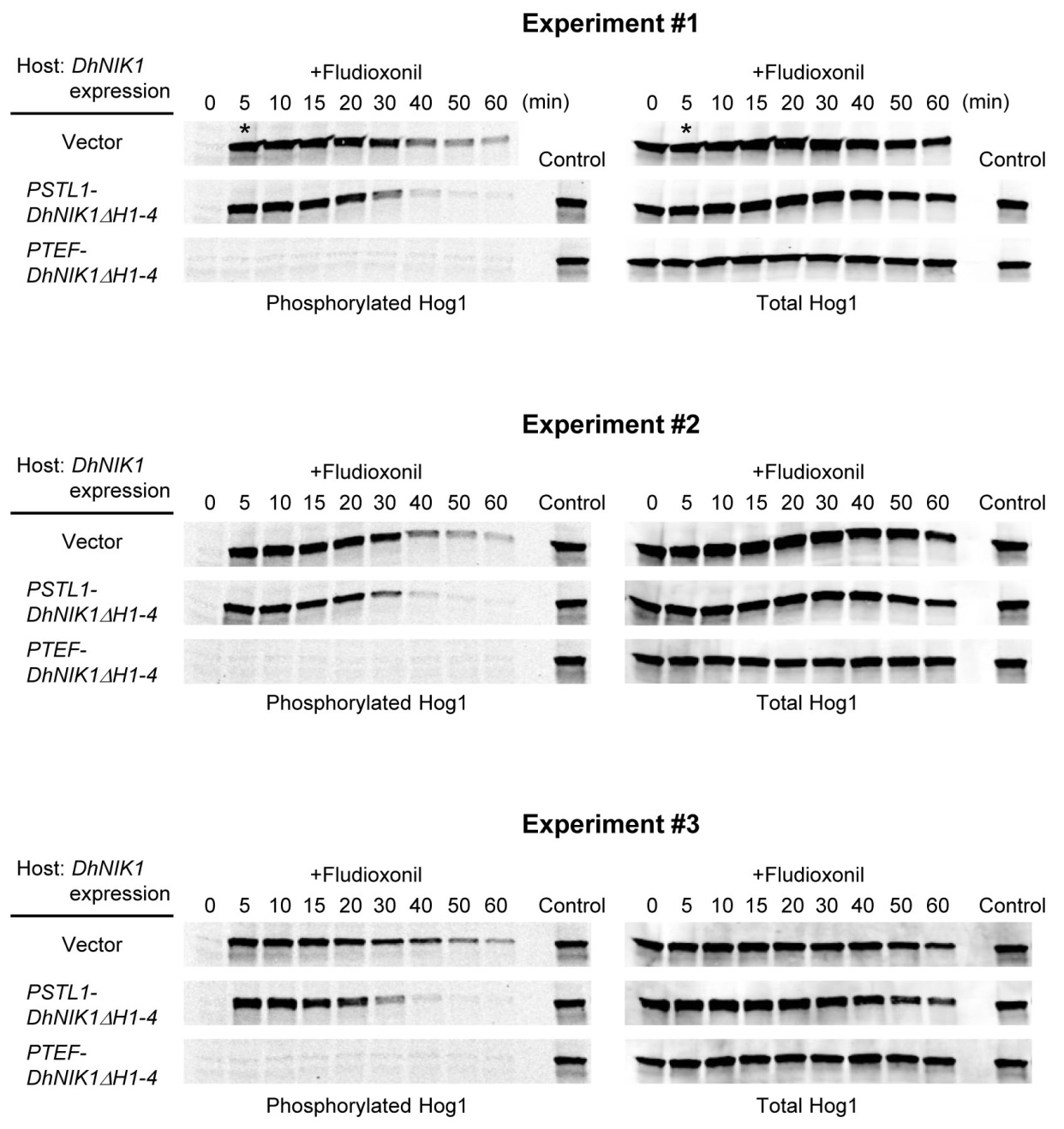


**Figure S2**


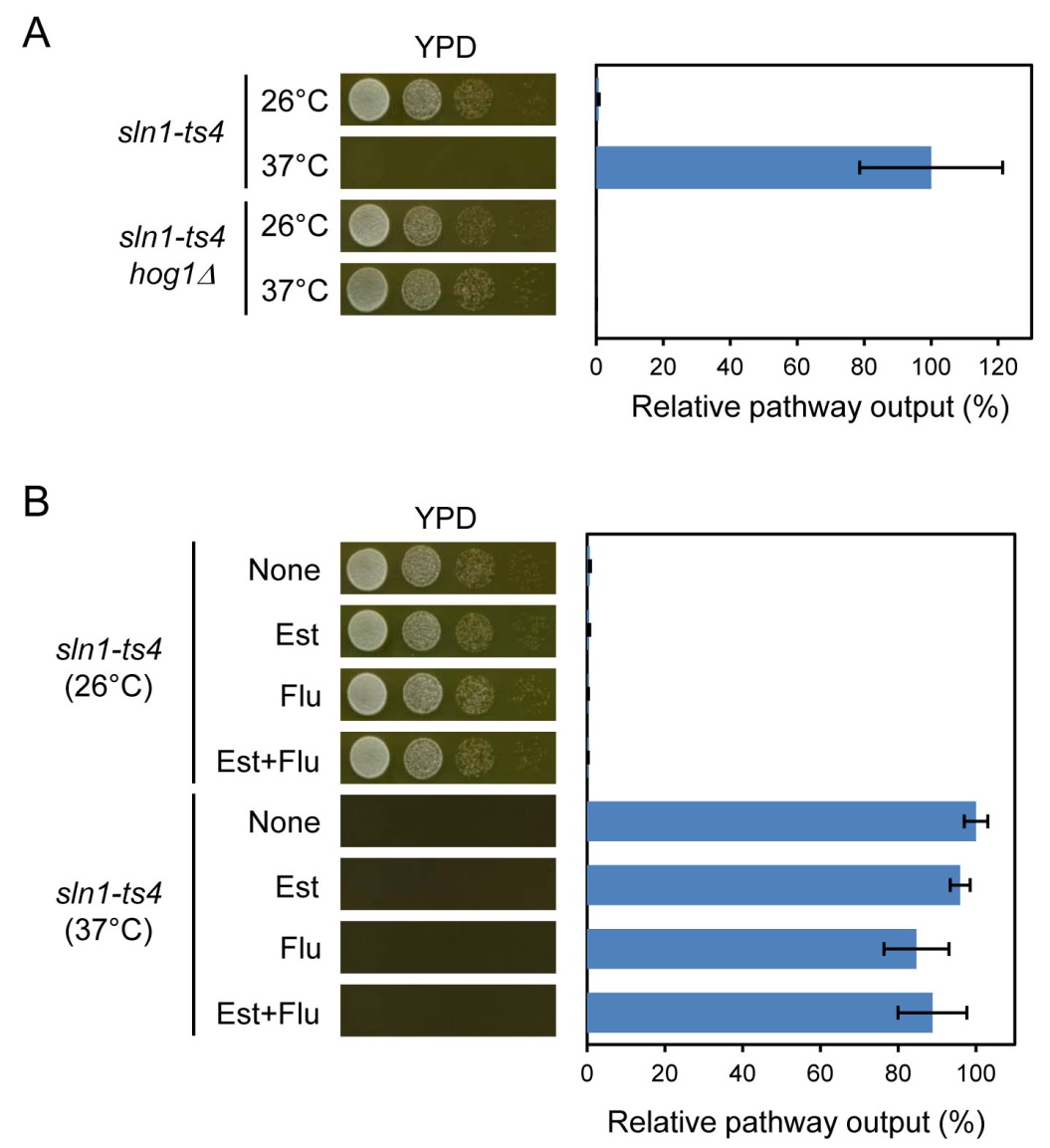


**Figure S3**


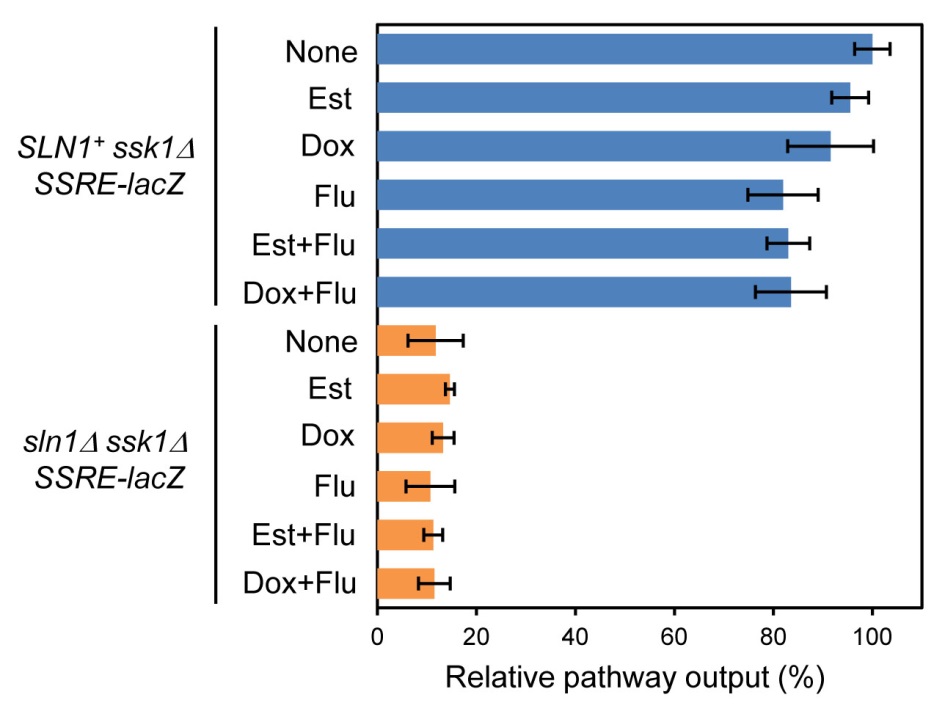


**Figure S4**


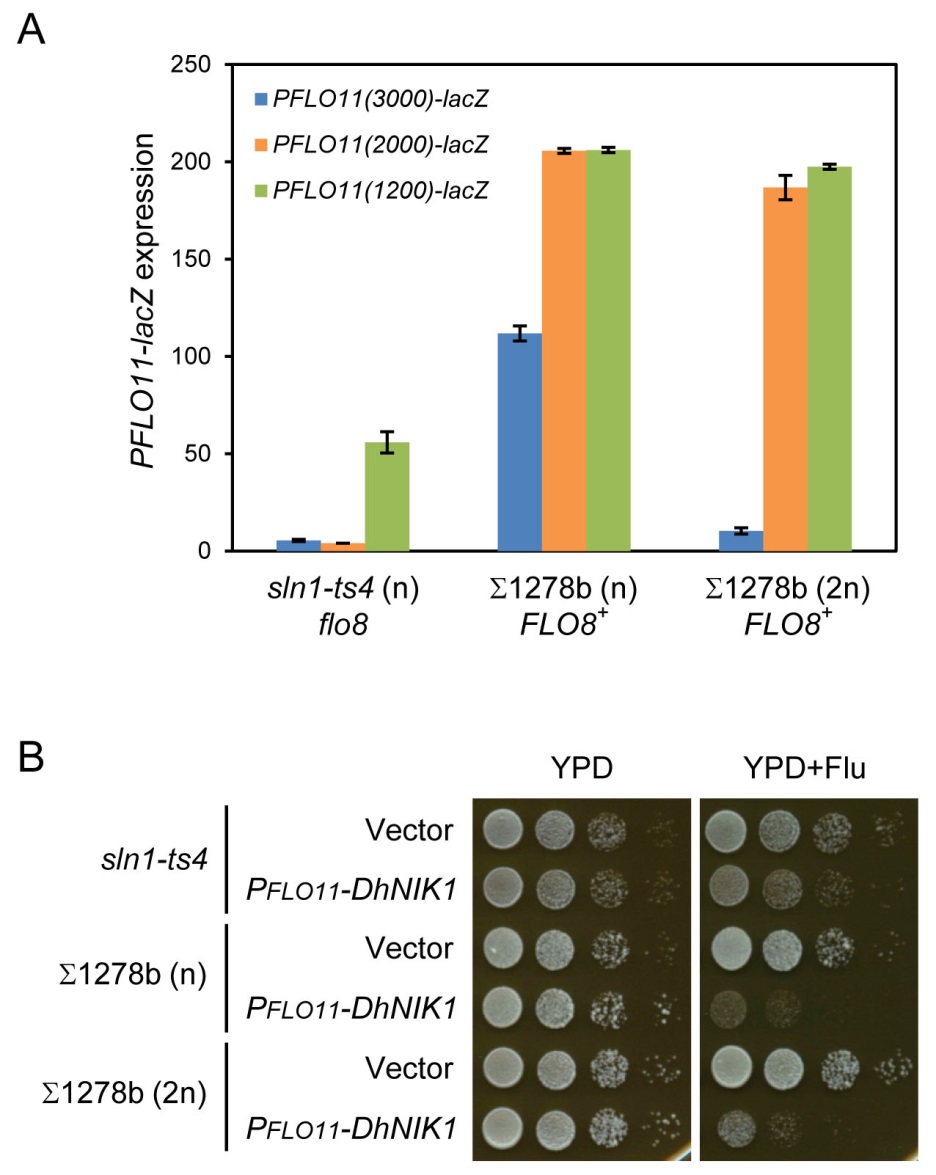

Supplement: SUPPLEMENTARY DATA [file supp_gkv678_nar-00779-h-2015-File007.docx]
